# Monoclonal antibodies against beta-amyloid protein (lecanemab and donanemab) should not be used in the treatment of Alzheimer's disease

**DOI:** 10.1055/s-0045-1808082

**Published:** 2025-05-09

**Authors:** Ricardo Nitrini, Adalberto Studart-Neto

**Affiliations:** 1 Universidade de São Paulo, Faculdade de Medicina, Hospital das Clínicas, Departamento de Neurologia, Grupo de Neurologia Cognitiva e do Comportamento, São Paulo SP, Brazil.

**Keywords:** Alzheimer Disease, Therapeutics, Antibodies, Monoclonal, Amyloid Cascade, Lecanemab, Donanemab, Minimum Clinically Important Difference

## Abstract

Two antiamyloid monoclonal antibodies (mAbs), lecanemab and donanemab, were recently launched for treatment of Alzheimer's disease (AD). These mAbs remove amyloid protein from the brain and cause statistically significant improvement in cognitive/functional tests, meaning a change in evolution of AD. This is important to reinforce the amyloid cascade hypothesis and to further concentrate studies on the pathways from the deposition of the beta-amyloid protein to synaptic loss and neuronal death. However, it is necessary to evaluate whether the results are clinically important. Analysis of the clinical trials showed that the statistically significant differences over placebo did not reach the minimum clinically important difference that would be meaningful for patients, caregivers and clinicians. Besides, the incidence of adverse events is high and potentially severe. Although there are reasons to celebrate this first step towards disease-modifying therapies for AD, lecanemab and donanemab should not be used to treat AD in clinical pratice.

*When you are studying any matter, or considering any philosophy, ask yourself only what are the facts and what is the truth that the facts bear out. Never let yourself be diverted either by what you wish to believe, or by what you think would have beneficent social effects if it were believed. But look only, and solely, at what are the facts.*

Bertrand Russell 1

## INTRODUCTION

In 1999, Shenk et al. reported that immunization with amyloid-beta peptide attenuated Alzheimer's-disease-like pathology in transgenic mice. 2 Subsequently, several trials with antiamyloid monoclonal antibodies (mAbs) were carried-out, without effects on clinical evolution. 3 4 These failures began to raise suspicions that the brain deposition of beta-amyloid peptide had no causal relationship with Alzheimer's disease (AD). 5

Recently, three antiamyloid mAbs have been approved by Food and Drug Administration (FDA) for AD treatment: aducanumab, lecanemab and donanemab. They have the common objective of removing beta-amyloid protein deposits from the brain parenchyma to change clinical evolution. 3 4 Aducanumab was withdrawn from the market by the pharmaceutical company in 2024. 3 Clinical trials with lecanemab and donanemab caused large reductions of brain deposits of beta-amyloid protein associated with statistically significant results in cognitive/functional tests. 6 7 8 The great importance of these clinical trials was to demonstrate the consistency of the amyloid cascade hypothesis, because removal of beta-amyloid protein deposits from the brain modified the evolution of AD. 3 4

These are important results to further concentrate studies in the pathophysiology of the cascade, from the deposition of the beta-amyloid protein to synaptic loss and neuronal death.

This first step towards disease-modifying therapies for AD was hailed by the media as positive results for AD treatment, raising expectations for the population. 3 4 However, it is still necessary to evaluate whether the results of these studies are clinically important.

## ASSESSMENT OF CLINICAL RESULTS

### Lecanemab

Lecanemab is a humanized mAb that binds with high affinity to soluble beta-amyloid protofibrils. 6 Its use was approved by the FDA in 2023, after publication of the results of the CLARITY AD clinical trial. 6 This trial enrolled 1705 participants with mild cognitive impairment (MCI) or mild dementia due to AD with a Clinical Dementia Rating (CDR) equal to 0.5 or 1.0. The primary outcome was the CDR sum of boxes (CDR-SB) score after 18 months of treatment ( Table 1 ). This score ranges from 0 to 18 points, with values from 0.5 to 6 indicating an early stage of AD. 6

**Table 1:** Baseline data summary from the CLARITY AD and TRAILBLAZER-ALZ 2 clinical trials

		CLARITY AD		TRAILBLAZER-ALZ 2*	
		Lecanemab (N = 859)	Placebo (N = 875)	Donanemab (N = 860)	Placebo (N = 976)
Mechanism of action		High affinity to soluble beta-amyloid protofibrils		High affinity to amyloid β found only in insoluble plaques	
Primary end point		CDR-SB (0–18)		iADRS (0–144)	
Global CDR (%)	0.0	0	0	0.2	0.5
	0.5	80.8	80.7	60.8	61.2
	1.0	19.2	19.3	36.0	35.4
	2.0	0	0	3.0	2.9
Mean CDR-SB (max. 18)		3.17 ± 1.34	3.22 ± 1.34	4.0 ± 2.1	3.9 ± 2.1
Mean MMSE score at baseline		25.5 ± 2.2	25.6 ± 2.2	22.4 ± 3.8	22.2 ± 3.9
Mean age (years)		71.4 ± 7.9	71.0 ± 7.8	73.0 ± 6.2	73.0 ± 6.2
*APOE ε4* allele (%)		68.9	68.6	69.8	71.2
Heterozygous		53.1	53.5	53.1	54.5
Homozygous		15.8	15.1	16.7	16.7

Abbreviations: APOE, apolipoprotein E gene; CDR, Clinical Dementia Rating; CDR-SB, Clinical Dementia Rating – Sum of Boxes Scale; iADRS; integrated Alzheimer Disease Rating Scale; MMSE, Mini-Mental State Examination. Note: *Combined tau group data.

At baseline, mean CDR-SB score was approximately 3.22 in lecanemab and placebo arms. 6 After 18 months, both groups had a mean decline of 1.21 and 1.66 points in CDR-SB, respectively, indicating a difference of only −0.45 (95% CI: −0.67 to −0.23; *p* < 0.001) in favor of lecanemab. 6 Although this is a statistically significant difference, there are doubts about its true clinical significance. 9 10 Andrews et al. estimated that the minimal clinically important differences (MCID) in AD clinical trials would be -0.98 and -1.63 points on the CDR-SB, for MCI and mild dementia, respectively. 9 Therefore, in CLARITY AD, lecanemab did not achieve this difference at the end of 18 months.

In subgroup analyses, lecanemab was not shown to be superior in women, black and Asian individuals, in patients younger than 65 years, and in homozygous carriers of the *APOE ε4* allele. 6 Some subgroups showed a favorable outcome for lecanemab with: male patients, those aged 75-years or older, and non-carriers of the APOE ε4 allele, although the CDR-SB mean difference between the two groups was only -0.73, -0.72, and -0.75 points, respectively. These values are lower than the MCID, as previously mentioned. 6 9 Although there is no consensus about these numbers, it is highly probable that, with a baseline of 3.22 points, a difference in the CDR-SB of -0.7 would not be perceived by the patient, physician or family members.

Furthermore, the subgroup of patients with MCI showed a smaller difference between lecanemab and placebo (-0.35; 95% CI: -0.56 to -0.14), compared to the subgroup with mild dementia (-0.62; 95% CI: -1.05 to -0.19). 6 11

### Donanemab

Donanemab is a humanized mAb that targets a form of beta-amyloid found only in plaques. 3 7 8 Its use was approved by the FDA in 2024, after the TRAILBLAZER-ALZ 2 study. 8

The primary outcome of this study was the evolution of scores on the integrated Alzheimer Disease Rating Scale (iADRS), an assessment of cognition and daily function, with a range from 0 to 144 points (lower scores indicate greater impairment). The MCID defined by the TRAILBLAZER-ALZ 2 was 5 points for MCI and 9 points for mild dementia. 8

Patients were divided into two groups: low/medium tau and combined (low/medium and high tau) population. At 76 weeks, brain amyloid plaque level decreased by 88.0 Centiloids (95% CI: -90.20 to -85.87) with donanemab and increased by 0.2 Centiloids (95% CI: -1.91–2.26) with placebo. 8 At the end of 76 weeks, the mean differences versus placebo in iADRS scores in combined groups were 2.92 (95% CI: 1.51–4.33), which was statistically significant, but did not reach the MCID defined by the study itself, as shown in Table 2 . There was no statistical difference between the evolution of the two groups of patients (low/medium tau population or combined low/medium and high tau population versus placebo). 8

**Table 2:** Summary of the main clinical end points and adverse events from CLARITY AD and TRAILBLAZER-ALZ 2 clinical trials

	CLARITY AD		TRAILBLAZER-ALZ 2*	
	Lecanemab (N = 859)	Placebo (N = 875)	Donanemab (N = 860)	Placebo (N = 976)
**Duration of follow-up**	18 months		76 weeks	
CDR-SB change follow-up (mean)**	1.21	1.66	1.72	2.42
Drug versus placebo (95% CI)	-0.45 (-0.67 to -0.23)		-0.70 (-0.95 to -0.45)	
iADRS change follow-up (mean)***	Not evaluated		-10.19	-13.11
Drug versus placebo (95% CI)			2.92 (1.51 − 4.33)	
**MMSE change follow-up (mean)**	Data not available		-2.75	-3.22
Drug versus placebo (95% CI)			0.48 (0.08 − 0.87)	
**Serious adverse events**	14.0	11.3	17.4	15.8
**Adverse events leading to discontinuation**	6.9	2.9	8.1	3.7
**Death related to treatment (%)**	0	0	0.4	0.1
**ARIA (%)**	21.5	9.5	36.8	14.9
ARIA-E (%)	12.6	1.7	24.0	2.1
Symptomatic ARIA-E (%)	2.8	0	6.1	0.1
Aria-E (%) in APOE e4 heterozygous	10.9	1.9	40.6	1.9
Aria-E (%) in APOE e4 homozygous	32.6	3.8	36.8	3.4
ARIA-H (%)	17.3	9.0	31.4	13.6
Symptomatic ARIA-H (%)	0.7	0.2		
Aria-H (%) in APOE e4 heterozygous	14.0	8.6	32.3	12.0
Aria-H (%) in APOE e4 homozygous	39.0	21.1	50.3	20.5

Abbreviations: APOE, apolipoprotein E gene; ARIA, amyloid-related neuroimaging abnormalities; CDR, Clinical Dementia Rating; CDR-SB, Clinical Dementia Rating – Sum of Boxes Scale; iADRS; integrated Alzheimer Disease Rating Scale; MMSE, Mini-Mental State Examination. Notes: *Combined tau group data; **Primary efficacy end point from CLARITY AD; ***Primary efficacy end point TRAILBLAZER-ALZ 2.

Secondary clinical objectives were achieved, but also did not reach values that could be considered MCID ( Table 2 ). In the mini-mental state examination (MMSE), mean differences with placebo were below 0.5. 8 As the minimal difference that could be detected by this tool is 1 point, donanemab would not cause perceptible change after 76 weeks.

The analysis of the evolution of scores in the different cognitive tests allows the conclusion that treatment with donanemab for 76 weeks had no meaningful effect.

### Minimum clinically important difference (MCID)

The need to better define MCID is fundamental to interpreting clinical trial outcomes and making clinical decisions. 9 10 Without these definitions, clinicians, patients, family members, caregivers, and health-care systems will continue to be guided by statistical significance that may indicate “only small and potentially inconsequential effects on clinical outcomes”. 10 In the TRAILBLAZER-ALZ 2, the differences against placebo in iADRS did not reach the MCID. As that was the primary outcome of the study, it is difficult to understand the approval of donanemab by the FDA. Recently, mathematical models have been presented to show possible effects of these treatments after the trials. 11 12 These extrapolations should be viewed very carefully, as they are not scientific evidence.

### Slowing of clinical disease progression (SCDP)

The results of SCDP were described as high for both lecanemab and donanemab. 6 8 For instance, there was 27.0% SCDP with lecanemab, for a difference in CDR-SB of -0.45. For a small difference of 2.92 in iADRS, SCDP was 22.3% with donanemab when compared to placebo. The calculation method may have influenced this dissociation, as it used the proportion of worsening with the drug versus placebo, 8 a very questionable method that does not take into account the magnitude or variance of the outcome measures. 13 14

More appropriate methods for evaluating the outcome of a treatment such as Cohen's d effect size, and number-needed-to-treat (NNT) were not used in these clinical trials. Goldberg et al. analyzed data from CLARITY AD and TRAILBLAZER-ALZ 2 study. 13

When the SCDP was described as 27.0% in CLARITY AD, for a difference in CDR-SB of -0.45, Cohen's d effect size was low (0.21), and NNT was 15. In TRAILBLAZER-ALZ 2, with a SCDP described as 29%, for a difference in CDR-SB of -0.7, Cohen's d effect size was also low (0.23), and NNT was 14. 13 It is relevant to remind that these differences in CDR-SB are below those estimated to be MCID. 9

Another way for evaluating SCDP is based on time savings in disease progression. This approach makes it easier to convey information about clinical results. 12 Nevertheless, time savings cannot be based solely on the proportion of worsening with the drug in relation to the placebo.

These clinical trials had as primary objectives changes in clinical biomarkers when compared to placebo. However, TRAILBLAZER-ALZ 2 also included changes in tau positron-emission tomography (PET) in frontal cortical regions when compared to placebo as secondary objective. There was no significant difference after 76 weeks. This is important because tau PET is now the main biomarker for biological staging of AD. 15

## DIFFERENCES BETWEEN EARLY AD AND RELATIVELY MORE ADVANCED AD CASES

In CLARITY AD, the difference between lecanemab and placebo in CDR-SB was lower in patients with MCI than in those with mild dementia. 6 In TRAILBLAZER-ALZ 2, there was no difference in the cognitive/functional evolution between the two groups. 8 These were unexpected findings because reducing amyloid deposits in early AD should be more efficient than in relatively more advanced cases. 16 However, “absence of evidence is not evidence of absence”. There is an ongoing trial using donanemab in 3,300 individuals with preclinical AD defined by unimpaired cognition and plasma biomarkers, which may be concluded in 2027 (TRAILBLAZER-ALZ 3). 17

## ADVERSE EVENTS

The incidence of adverse events was high both in the CLARITY AD study and in TRAILBLAZER-ALZ 2 ( Table 2 ). The most serious complication, which has been called amyloid-related imaging abnormalities (ARIA), characterized by edema/effusion (ARIA-E), or microhemorrhages and hemosiderin deposits (ARIA-H). Incidence of ARIA-H was high with both mAbs, 6 8 reaching 31.4% with donanemab. It was also high with placebo ( Table 2 ). The incidence of ARIA was higher in *APOE ε4* allele carriers. 6 8 Although most of these adverse events were considered mild to moderate, three patients receiving donanemab with serious ARIA subsequently died. 8 There were no deaths during the CLARITY AD study, but an extension study had nine, four of which were possibly related to the use of lecanemab. 18 Discontinuation due to adverse events was more than twice as frequent in participants who received these treatments than in placebo ( Table 2 ). 6 8

Two findings are relevant here:

- the high incidence of microhemorrhages and hemosiderin deposits in patients receiving antiamyloid mAbs; and
- the smaller but also high incidence of microhemorrhages and hemosiderin deposits in the placebo group.

These facts indicate that AD patients have a higher risk of spontaneous microhemorrhages, probably due to amyloid angiopathy. 19 20 Antiamyloid mAbs probably increase this risk by their effects on the vessel walls already affected by amyloid angiopathy.

## PATIENT SELECTION TO RECEIVE TREATMENT

When the patient and care-partner are willing to receive these treatments after receiving all this information, only a small proportion of patients will be eligible to be treated, due to several restrictions to their use. In a study, only 12 out of 237 volunteer participants with MCI or mild dementia due to AD would be selected to receive lecanemab after applying the clinical trial's inclusion and exclusion criteria. 21

In conclusion, antiamyloid mAbs change the evolution of AD and reinforce the importance of amyloid cascade hypothesis. However, lecanemab and donanemab should not be used in the treatment of AD because cognitive/functional markers did not achieve MCID, whereas the adverse events are high and potentially severe. Also, there were no differences in cognitive/functional biomarkers between early AD and mild dementia with these two mAbs.

Treating very early AD would probably be more successful, as is the case in oncology. However, it was not proven for these two mAbs. Using them to treat very early manifestations of AD would not be based on available scientific evidence, that is, when positive effects are higher than adverse events. Future clinical treatments may include improved immunotherapies together with treatments targeting other steps in the pathophysiology of AD. Probably treatments will start earlier. Future clinical trials shall include MCID, Cohen's d effect size, NNT or time saving calculation (or even better methods to analyze effect size).
